# Effects of Two Salts Compounds on Mycelial Growth, Sporulation, and Spore Germination of Six Isolates of *Botrytis cinerea* in the Western North of Algeria

**DOI:** 10.1155/2015/572626

**Published:** 2015-03-26

**Authors:** Boualem Boumaaza, Mohamed Benkhelifa, Moulay Belkhoudja

**Affiliations:** ^1^Department of Agronomy, Laboratory of Plant Protection, University of Abdelhamid Ibn Badis, BP 300, 27000 Mostaganem, Algeria; ^2^Sciences Faculty, Vegetal Ecophysiology Laboratory, University of Es Senia, BP 1524, ElMnouer, Oran, Algeria

## Abstract

Six isolates of *Botrytis cinerea* were isolated from leaves and stems of different tomato varieties taken from four areas in the northwest of Algeria where tomato is mostly grown in greenhouses and high tunnels. The purpose of this research was to determine the effect of two salts, NaCl and CaCl_2_, on three stages of *Botrytis cinerea's* life cycle. All isolates tested were stimulated in 50 to 150 ppm; NaCl was the most effective treatment to increase mycelial growth at two tested concentrations. However, at 300 ppm concentration, CaCl_2_ completely inhibited the growth of mycelium; they reach 34.78% for the isolate TR46 and 26.72% for isolate F27. The sodium and calcium salts stimulated conidia production in liquid culture. We noticed that the effect of calcium chloride on sporulation was average while sodium chloride. In the medium containing 50 ppm, calcium chloride and sodium chloride increased the germination capacity of most isolates compared with the control. Other calcium salts, at 100 or 300 ppm, decreased the germination percentage of the conidia. With the exception of sodium salts, the inhibitions of germination reduce at 150 or 300 compared with the control. Conidial germination was slightly inhibited by sodium chloride only when the concentration was over 300 ppm.

## 1. Introduction

Grey mould, caused by* Botrytis cinerea* (Sclerotiniaceae family), is an important plant disease that affects a large number of plant species and is particularly important in greenhouse production of tomatoes in the Mediterranean Basin [1]. In greenhouse tomato, the fungus infects flowers, fruits, and leaves and can grow through the petiole into the stem [2, 3].

Soil salinity is a major constraint to agricultural production around the world. This problem is one of the major stresses especially in arid and semiarid regions [4] and can severely limit plant growth and productivity [5, 6].

In Algeria, a wide range of environmental stresses (such as high and low temperature, drought, alkalinity, salinity, and pathogen infection) are potentially harmful to the plants. Soil salinity and irrigation water are two of the main serious problems hindering the development of most plant species [7]. Thus, the effect of these factors may result from structural and physiological changes in the plant, an increased incidence, and severity of diseases caused by various species pathogen. Reference [8] showed that relatively low levels of salinity (25–50 mEq) could increase the severity of* Phytophthora* root rot of tomato with high Na : Ca ratios (10 : 1),* Phytophthora* [9–12],* F. oxysporum f.* sp.* vasinfectum* [13],* F. oxysporum f.* sp.* radicis*-*lycopersici* [14], and* Verticillium dahliae* and* Alternaria solani* [15].

Ca is an essential element in all plants [16]. The ability of Ca to form intermolecular linkages gives it an important role in maintaining the integrity and structure of membranes and cell walls [17]. Ca is also used as a second messenger in many signal transduction pathways within the cell [18]. Its role in the physiology of plant tissue is well established [19]. Several studies have reported that Ca treatment of plant tissue induces an increase in tissue Ca content [20], resulting in reduced fungal diseases. The mechanisms by which calcium salts inhibit the development and severity of diseases are not known. One hypothesis is that high external Ca^2+^ concentrations may increase the concentration of Ca^2+^ in the cytosol, which can be toxic to the fungus. The ability of calcium to reduce the development of postharvest diseases of fruit has been attributed mainly to the formation of calcium cross-linkages in the cell wall, resulting in decreased effectiveness of cell wall-macerating enzymes secreted by the pathogen [21]. Reference [22] also demonstrated a relationship between increasing levels of calcium in the cell walls of potato tubers and a reduction in the macerating activity of* Erwinia carotovora*.

All the studies on the effect of Ca on* Botrytis* showed that it has an inhibitory effect on growth of this fungus at high concentrations [23–25]. This effect is thought to be mainly due to the role of calcium in ameliorating physiological disorders and thus indirectly reducing pathogen activity [26, 27]. Reference [25] has indicated that calcium chloride reduced germination and germ tube elongation* of B. cinerea* and* Penicillium expansum* in vitro. Reference [28] also has reported a similar effect of calcium in reducing the susceptibility of rose flowers to gray mold caused by* Botrytis cinerea*.

For the most effective control of disease, it seems necessary to examine the impact of salinity on the development of pathogen. The objective of this study was to determine the in vitro effect of sodium and calcium salts on spore production, conidia germination, and mycelial growth of* B. cinerea*.

## 2. Materials and Methods

### 2.1. Fungal Isolates

*B. cinerea* isolates were obtained from decayed tomato (*Lycopersicon esculentum*) in northwestern Algeria. The leaf fragments were placed on filter paper moistened with sterile water in a Petri dish.* B. cinerea* was cultured on potato dextrose agar (PDA) incubated at 25°C. Conidia were harvested from 14-day-old cultures by agitating small pieces of agar, bearing mycelia and conidia, in a glass tube.

### 2.2. Effect on Sporulation

Conidia of* B. cinerea* were obtained from 2 week old PDA cultures incubated at 25°C in 12/12 hours light/dark. Culture plates were vortexed in a tube containing 10 mL sterile distilled water and 0.05 mL Tween 80. A sterile magnetic stir bar was placed on the agar and set stirring for 5 minutes to loosen the spores. The suspension enriched in spores was filtered through glass filter to eliminate mycelium. Finally the conidial concentration was determined using a Malassez cell and adjusted to 10^5^ spores per mL.

### 2.3. Effect on the Germination of Conidia

To determine the influence of NaCl and CaCl_2_ on spore germination of* B. cinerea*, a drop containing 100 conidia was transferred onto water agar plates enriched with NaCl and CaCl_2_: 0, 50, 100, 150, and 300 mEq. The plates were incubated at 25°C in the dark for 24 hours. Results were expressed as the percentage of germinated conidia. A conidium was considered as germinated if the germ tube length was at least twice the length of the conidium.

### 2.4. Effect on Mycelial Growth

The influence of NaCl and CaCl_2_ on the diameter growth was determined by growing the isolates in a PDA medium at four NaCl and CaCl_2_ levels (50, 100, 150, and 300 mEq); control medium was not amended with salts. Mycelium growth inhibition was evaluated by placing a plug (4 mm diameter) from an actively growing culture in the centre of a PDA agar plate of 9 cm plastic Petri dishes. Cultures were incubated for 7–14 days at 25°C in the dark, and each treatment had four replications. Colony diameters were measured at two perpendicular directions.

### 2.5. Statistical Analysis

All statistical analyses were analyzed by the software of statistics (STATBOX 6.0.4, Grimmersoft). The data were analyzed by two-way factorial. Comparison of means and interactions was performed by Duncan's multiple range tests. Statistical significance was assessed at the level of *P* = 0.05 or *P* = 0.01.

## 3. Results

**Figure 1:**
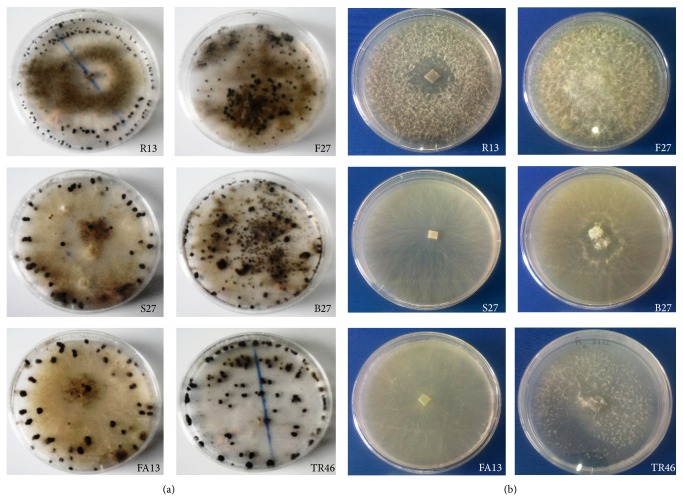
Morphological types of* B. cinerea*. (b) Mycelial growth on potato dextrose agar (PDA) medium at 25°C after two weeks. (a) Distribution and pattern of sclerotia formation on the surface of PDA after 4 weeks.

*Morphological Characteristics*. All the isolates exhibited variation in their colony characteristics such as color, shape, and texture (Figure 1).

*B. cinerea* colonies from tomato on PDA at 25°C were visually classified into three morphological groups, GI, GII, and GIII, based on colony color and pycnidial distribution. GI (F27) isolate produced white to light grey colonies, where colony texture was generally cottony, and was present at the center of the Petri dish. GII (FA13, S27, B27, and TR13) isolates had off-white to pale gray color. Colonies generally had a medium texture. GIII (R13) isolate had grown and spread rapidly by aerial mycelia growth. The colony was generally grey color. Based on the morphological parameter of length, pycnidia can be classified into two groups: length greater in isolates FA13, S27, B27, and F27 and length less in isolates TR13 and R13. Sclerotia also vary in abundance and distribution. In some isolates both superficial and imbedded sclerotia were produced. They scattered all over the medium in Petri dish, covering the entire surface of the agar (B27 and TR13 and F27). In some isolates sclerotia were produced on concentric rings, formed along the edges of the Petri dish (FA13, S27, and R13). Sclerotia were variable in shape and size. They were black, rounded, roughly circular, or irregular in shape.

### 3.1. The Effects of NaCl and CaCl_2_ of Culture Medium on Mycelial Growth of Six Isolates

This study evaluated the activity of 2 salts against* B. cinerea* in vitro at 4 concentrations. The effect of salts at different concentrations on mycelial growth of colonies of six isolates of* B. cinerea* in PDA was observed after 3 days. The results are shown in Table 1. All concentrations, except 300 ppm, of NaCl significantly stimulated growth of* B. cinerea.* (*P* < 0.001). Beyond this range, reduced growth has been correlated with the increasing in the NaCl of the medium.

The calcium chloride at 50 and 100 ppm stimulated mycelial growth of* B. cinerea* relative to the control. However, higher concentrations of calcium chloride (150 and 300 ppm) caused F27, B27, R13, TR46, S27, and FA13 to reduce growth by 26.72, 24.26, 23.24, 34.78, 15.07, and 17.04%, respectively. There was a significant reduction in growth of isolates (*P* < 0.001) with increasing calcium salt. At 300 ppm, calcium chloride was the most inhibitory, reducing growth on PDA by 34.78% for the isolate. The interaction between salt and concentration was significant for R13, TR45, S27, and FA13 (*P* < 0.001), but not significant for F27 and B27 isolates.

All concentrations were significantly different from the control (*P* < 0.001); 150 ppm CaCl_2_ and 300 ppm NaCl or CaCl_2_ were similar to each other and different from other concentrations in reducing mycelial growth.

### 3.2. The Effects of NaCl and CaCl_2_ on the Production of Spores of the Six Isolates

In the absence of salt (Table 2), isolates of* B. cinerea* do not present the same profile of conidial production.

The optimum density for spore production of this fungus was from 1.97 10^6^ to 2.2 10^6^ spores/mL, of R13 and B27, respectively. Data in Table 2 indicate that application of chloride salts and sodium salt caused a significant increase in the production of conidiogenesis at various concentrations tested compared with control (*P* < 0.001). However, these observations indicate that the conidial production of the six isolates might increase, even at high salinity. There was a significant increase in these characters between the control and 50 ppm concentration of salinity.

Under these salt conditions, sodium chloride is most favorable to the sporulation of all isolates of* B. cinerea*, especially at high concentrations of the culture medium. By adding 300 ppm of NaCl to the culture medium, an increase in spore production by 1.21 10^7^ spores/mL can be obtained from the isolate B27. Calcium chloride stimulates little sporulation of the isolates, with only 9.2 10^6^/mL to isolate R13. The amount of spore production is in direct proportion to the concentration of salinity in the culture medium. The interaction between salt and concentration was significant for all salts isolates (*P* < 0.05) except FA13 (Table 2).

The results for the percentage germination of different isolates studied in terms of germination capacity under the effect of different salt concentrations are shown in Table 3.

The six isolates presented dissimilar percentages of germination in the presence of sodium and calcium chloride. The analysis shows that salinity affects the percentage of germination for each value of salt. There are significant differences between saline treatments. The highest germination percentage, 78.33% and 63.67%, was obtained in the absence of salt after 24 h incubation at 25°C, from isolates FA13 and TR46, respectively. Concerning the salts, the 50 and 100 ppm also increased the conidial germination except TR13 and FA13 isolates, which was significantly different from the stimulation caused by NaCl. Conversely, spore germination was decreased for sodium concentrations of 150 and 300 ppm relative to the control.

The application of lower concentrations of CaCl_2_ (50 ppm) to wounds did not inhibit their percentage germination. However, germination percentages were very low in all concentrations except 50 ppm. CaCl_2_ was toxic at lower concentrations than the other salts. The toxicity of calcium chloride (EC50 = 100 ppm) to spores was higher than that of sodium chloride (EC50 = 150 ppm). The interaction between salt and concentration was significant for all isolates (*P* < 0.001) except F27 and TR46.

## 4. Discussion

The purpose of this study was to compare the effect of sodium and calcium salts against* B. cinerea*. Our in vitro tests showed that sodium chloride stimulates the development of the fungal up to 150 ppm. In contrast, only calcium salts were effective at low concentrations as compared to sodium chloride. However, higher concentrations of calcium chloride reduced mycelial growth of* B. cinerea* PDA medium.

Our data indicate that salinity stimulated growth of all six isolates at high concentration (NaCl at up to 150 ppm). On the contrary, high salinity (more than 300 ppm) of several sodium has been shown to decrease mycelial growth and inhibit spore germination. Reference [29] showed that increasing the salinity of the medium promotes the in vitro mycelial growth of* Phytophthora citrophthora* and* P. parasitica*, agents of root rot of citrus, with an optimum between −1.44 and −3.11 bars. Similarly, [23] showed that all calcium salts tested (except formate, calcium pantothenate, and dibasic calcium phosphate) reduced the growth of* Monilia fructicola* responsible for brown rot of peach, on amended potato dextrose agar (PDA).

In the comparison of the inhibitory effect of the various salts, higher concentrations with 300 ppm to NaCl or CaCl_2_ reduced mycelial growth to 16 and 23%, respectively. The mechanisms by which sodium salts effect mycelia growth are not known. Regragui and Lahlou [30] showed that the stimulator effect of salinity was observed on the mycelial growth, conidia production, and conidia germination of the tested stain of* V. dahliae*, respectively, in concentrations 170, 120, and 256 mM of NaCl. Oppositely, Pelizza et al. [31] showed that the presence of NaCl in the medium culture reduces the growth of an isolate of* Leptolegnia chapmanii*. However, Reid et al. [32] reported that sodium chloride was more effective than other chloride salts (calcium chloride, ammonium chloride, and manganese chloride) in controlling* Fusarium* crown and root rot caused by* F. oxysporum f.* sp.* asparagi* and* F. proliferatum*. van Bruggen and Semenov [33] reported that on a long-term basis there is a decrease in the genetic diversity of fungi as a result of stress. On the other hand Zahran [34] mentioned that the hydric stress has to deal with the increase in osmotic pressure and may therefore change their physiology [35] and morphology in response to this [34]. Two strategies used by microorganisms to adapt to osmotic stress were described by Killham [35], both of which result in an accumulation of solutes in the cell to counteract the increase in osmotic pressure. One is the selective exclusion of the solute incorporated (e.g., Na^+^, Cl^−^), thus accumulating the ions necessary for metabolism (e.g., NH_4_^+^).

The results of the present study demonstrate that calcium salts also have been shown to reduce mycelial growth in vitro; the percentage of reduction varied between 15 and 34% as compared to the control. Several studies have reported that calcium applications can suppress diseases caused by several pathogens [23, 27, 28, 36]. Our result further supports the results of Maouni et al. [37], who found that calcium chloride significantly reduced pear fruit decay caused by* A. alternata* and* Penicillium expansum* when used at 4 and 6%. Tian et al. [38] recorded that calcium chloride at 2% inhibited the growth and spore germination of* R. stolonifer*, although CaCl_2_ was tolerated by* Alternaria alternata* and* P. expansum* in vitro. It was reported that 1,000 mg of calcium (calcium chloride) enhanced the growth of* Botryosphaeria dothidea* [39]. Calcium salts also have been shown to reduce mycelial growth in vitro and reduce incidence and severity of infection of peach fruits and shoots by* Monilinia fructicola* and* Leucostoma persoonii*, respectively [23, 40]. Kaile et al. [24] reported that mycelia cultured with 100 to 200 mM Ca^2+^ exhibited a lower viability compared with mycelia grown with 10 mM Ca for some isolates of* Botrytis* spp. While little information is available on the role of Ca^2+^ in fungi, results of experiments with yeasts have shown that mutants that have defective intracellular Ca^2+^ transport systems or defective vacuolar H^+^-ATPase that produces the proton motive force necessary for the activity of the vacuolar Ca^2+^/H^+^ exchanger [41] could not grow in high Ca^2+^ concentrations [42–45]. Maintenance of low basal concentrations of free cytosolic Ca^2+^, in the submicromolar range, is essential for normal cell functions [46, 47].

In the case of the evaluation the effect of NaCl and CaCl_2_ on the spore production by the fungus, all isolates of* B. cinerea* are able to sporulate in salinity tested, but to varying degrees. The largest sporulation was observed for different concentrations between 50 and 300 ppm. The incorporation of 300 ppm salt stimulated the sporulation. In fact, sporangium formation of* Phytophthora parasitica* in vitro appeared to be stimulated by salinity, as the numbers of sporangia were generally higher (120% to 225%) in the salt-amended treatments than the distilled water controls [30]. With regard to conidiogenesis, sodium chloride was significantly higher than those at the other calcium chloride concentrations. Reference [25] showed that stimulation of sporulation under the effect of salinity is due to a specific effect of ions. According to this author, Na^+^ and Cl^−^ stimulate the production of sporangia of* P. citrophthora* and* P. parasitica* while the osmotic effect inhibits biological activity. In* Verticillium*, increased sporulation under the effect of the salt appears to be not only solely due to the effect of Na^+^ and Cl^−^ ions, but also due to the osmotic effect. However calcium salts did not reduce spore production of* B. cinerea* spores in this study. Similarly, a minimum concentration of calcium is necessary for the production of zoosporangia or zoospore release by* Phytophthora* spp. [48–50].

In view of these findings, it was reported that, at low concentration (50 ppm), the germination capacity for most isolates increased compared with the control in both types of salt. Beyond this concentration, the effect of CaCl_2_ resulted in a linear reduction in % of germination. Similarly, a low reduction of conidial germination was observed for two salt types at the maximum concentration used. Reference [42] showed that increasing the concentration of calcium chloride (25–175 mM) causes a decrease in germination and germ tube growth in vitro of* B. cinerea* and* Penicillium expansum*, respectively, causing the gray and blue mold in apples stored. Incubating* B. cinerea* spores in increasing concentrations of CaCl_2_ (4–26 g L^−1^) resulted in decreased spore germination and germ tube growth [51]. Calcium was effective in inhibiting spore germination of* C. gloeosporioides* [23],* Rhizopus stolonifer* [37], and* Alternaria alternata* and* Penicillium expansum* [52]. Physiologically, the maintenance of low basal concentrations of internal Ca^2+^ is essential for normal cell functions of organisms, and the inability to regulate Ca^2+^ may affect the organisms' normal growth [53].

Calcium ions may reduce the incidence of fungal infection by directly inhibiting fungal growth and by inhibiting cell wall degrading enzymes produced by the pathogens [45, 54, 55].

The effects of calcium in reducing spore germination were probably due to toxicity, with high concentrations likely affecting the osmotic balance in fungal cells [56].

## Figures and Tables

**Table 1:** Effect of different concentrations of NaCl and CaCl_2_ on the percentage of mycelial growth areas (millimeter) of six isolates of *Botrytis cinerea* in PDA medium after incubation at 25°C for 3 days.

Treatment	*B*. *cinerea* isolate					
	F27	B27	R13	TR46	S27	FA13
Control	36.16^a^	38.5^c^	40.16^c^	38.33^b^	31^cd^	37.16^cd^
NaCl						
50	37.83^ab^	42^b^	41.5^bc^	41.2^a^	35.16^ab^	38.66^abc^
100	38.5^a^	44.33^a^	45.5^a^	43.5^a^	37.83^a^	40.33^a^
150	38^a^	41.5^c^	44^ab^	43^a^	37.66^a^	39.83^ab^
300	31.16^d^	31.33^d^	32.16^de^	32.66^d^	28.33^de^	28^f^
CaCl_2_						
50	36.5^a^	40.5^b^	41.33^bc^	40.83^ab^	32.66^bc^	38.33^bc^
100	37^d^	42^a^	42.5^bc^	42.5^a^	34.16^bc^	39.5^ab^
150	33.5^a^	37.33^a^	34^d^	35.5^c^	28.66^de^	36^d^
300	26.5^e^	29.16^c^	30.83^e^	25^e^	26.33^e^	30.83^e^
Two-way factor analysis of variance						
Salt	*P* = 001	*P* = 00018	*P* = 0.00001	*P* = 0	*P* = 0.00001	*P* = 0
Concentration	*P* = 0	*P* = 0	*P* = 0	*P* = 0	*P* = 0	NS
Salt ∗ concentration	NS	NS	*P* = 0.0001	*P* = 0.00001	*P* = 0.001	*P* = 00004

*P* = probability value (significance level). NS; not significant. Z: Letters denote significant differences among means in columns within trial according.

**Table 2:** Effect of different concentrations of NaCl and CaCl_2_ on sporangium formation by six isolates of *Botrytis cinerea* in vitro after 14 days in potato dextrose agar (PDA) at 25°C.

Treatment	*B*. *cinerea* isolate					
	F27	B27	R13	TR46	S27	FA13
Control	14.25^c^	22.7^c^	19.75^d^	20.2^b^	10.37^d^	8.87^d^
NaCl						
50	26.49^c^	29.55^c^	21.16^d^	22.57^b^	20.39^c^	21.1^c^
100	31.33^bc^	31.33^c^	45.54^c^	35.62^b^	32.04^d^	29.58^b^
150	63.04^b^	57.62^b^	88.41^b^	41.87^b^	68.65^b^	35.66^b^
300	109.25^a^	121.45^a^	107.49^b^	95.83^b^	95.53^a^	56.11^a^
CaCl_2_						
50	27.29^c^	26.96^c^	24.74^d^	21.29^b^	34.5^c^	15.95^d^
100	27.62^c^	32.41^c^	28.19^cd^	24.33^b^	31.54^c^	28.16^c^
150	35.33^bc^	36.36^c^	45.66^c^	28.74^b^	46.66^c^	35.33^b^
300	59.16^b^	67.47^b^	90.29^a^	54.37^a^	73.7^b^	59^a^
Two-way factor analysis of variance						
Salt	*P* = 0.0005^z^	*P* = 0.0008	*P* = 0.024	NS	*P* = 0.034	NS
Concentration	*P* = 0	*P* = 0	*P* = 0	*P* = 0.00002	*P* = 0	*P* = 0
Salt ∗ concentration	*P* = 0.008	*P* = 0.00009	*P* = 0.00001	*P* = 0.029	*P* = 000018	NS

*P* = probability value (significance level). NS; not significant. Z: Letters denote significant differences among means in columns within trial according.

**Table 3:** The effect of sodium and chloride salts on the germination of Six Isolates of *Botrytis cinerea* (percent germination was measured ater 24 h incubation at 25°C).

Treatment	*B. cinerea* isolate					
	F27	B27	R13	TR46	S27	FA13
Control	54^b^	56^b^	58.66^ab^	63.67^a^	50.33^d^	78.33^b^
NaCl						
50	69^a^	67.33^a^	61.66^ab^	64.66^a^	66.66^a^	90.66^a^
100	66.33^a^	68.33^a^	60.66^ab^	32.33^b^	61.33^b^	63.33^c^
150	50.33^b^	52^b^	34.33^c^	26.33^b^	57.33^c^	61.66^c^
300	41^c^	33.33^c^	32.33^c^	23^b^	39.33^e^	42.66^e^
CaCl_2_						
50	58^a^	60.33^ab^	69.66^a^	65.33^a^	55^c^	88^a^
100	50^ab^	51^b^	55.33^ab^	39.66^b^	41.33^e^	49.66^d^
150	45^b^	29.33^c^	50.66^b^	35.66^b^	36.33^f^	38.33^e^
300	33.33^c^	25.66^c^	18.29^d^	30.33^b^	18.33^g^	28.33^f^
Two-way factor analysis of variance						
Salt	*P* = 0.00001^z^	*P* = 0	NS	*P* = 0.04	*P* = 0	*P* = 0
Concentration	*P* = 0	*P* = 0	*P* = 0	*P* = 0	*P* = 0	*P* = 0
Salt ∗ concentration	NS	*P* = 0.001	*P* = 0.0007	NS	*P* = 0	*P* = 0.0003

*P* = probability value (significance level). NS; not significant. Z: Letters denote significant differences among means in columns within trial according.
